# *Idobata-Nagaya*: a community housing solution for socially isolated older adults following the great East Japan earthquake

**DOI:** 10.3389/fpubh.2023.1289552

**Published:** 2023-11-22

**Authors:** Toshiki Abe, Hiroaki Saito, Nobuaki Moriyama, Naomi Ito, Morihito Takita, Yuri Kinoshita, Akihiko Ozaki, Yoshitaka Nishikawa, Chika Yamamoto, Tianchen Zhao, Mika Sato, Masaharu Tsubokura

**Affiliations:** ^1^Department of Radiation Health Management, Fukushima Medical University School of Medicine, Fukushima, Japan; ^2^Department of Internal Medicine, Soma Central Hospital, Fukushima, Japan; ^3^Department of Public Health, Fukushima Medical University School of Medicine, Fukushima, Japan; ^4^Research Division, Medical Governance Research Institute, Tokyo, Japan; ^5^Division of Food Science and Nutrition, Tohoku Seikatsu Bunka Junior College, Sendai, Miyagi, Japan; ^6^Department of Breast Surgery, Jyoban Hospital of Tokiwa Foundation, Fukushima, Japan; ^7^Department of Internal Medicine, Hirata Central Hospital, Fukushima, Japan; ^8^Department of Health Nursing of International Radiation Exposure, School of Medicine, Fukushima Medical University, Fukushima, Japan

**Keywords:** social isolation, disaster, collective housing, long-term care, preventive care

## Abstract

**Introduction:**

Following the Great East Japan Earthquake, the living environment of socially isolated older adults has become a pressing concern. In response, *Nagaya*, a collective housing program, was established in Soma City, Fukushima, Japan to address social isolation among older adults and support their long-term health. This study aimed to identify characteristics of individuals in Nagaya and examine the sustainability of this initiative.

**Methods:**

We conducted a retrospective analysis of residents who were relocated to *Nagaya*, emphasizing their characteristics, the continuity of their stay in *Nagaya*, and their care certification levels, using data up to December 31, 2022. We employed Kaplan–Meier curves to analyze the duration for which residents continued to reside in *Nagaya* and the time leading up to the requiring care-level certification.

**Results:**

Of 65 people who moved to *Nagaya* after the disaster, 30 people (46.2%) continued to live there, 21 (32.3%) died during their stay, and 14 (21.5%) moved out. The overall duration of occupancy averaged 6.39 years (SD 3.83 years). The proportion of requiring care-level certification occurrences per person-year was 0.0577 for those without care certification and 0.3358 for those with requiring support level at the time of moving in.

**Conclusion:**

In summary, *Nagaya*-style communal housing may offer suitable living environments for older adults with diverse needs during disasters and serve as a valuable tool for developing public policies in aging societies.

## 1 Introduction

Promoting a longer, healthier life expectancy for older people is becoming increasingly important in regions with rapidly aging populations (1). In addition to various factors, including healthy diets, regular physical activity (2, 3), and economic stability (4), social factors, such as social interaction (5), community involvement (6), and the environment to live according to their own will (7), contribute to healthy living. However, older adults at high risk of social isolation might struggle to create such environments independently. Consequently, families and local governments need to provide supportive environments for older adults (8, 9).

In developed countries with rapidly aging populations, addressing the health, housing, and caregiving concerns of socially isolated older adults is becoming a critical public health issue. In 2020, Japan had an aging rate of 28.6%, with 15.0% of older men and 22.1% of older women living alone (10). China is also undergoing unprecedented rapid aging among developed nations (11). Along with population aging, social isolation and loneliness are increasingly recognized as significant health problems (12). Numerous studies have demonstrated that social isolation and loneliness can worsen the prognosis of affected individuals and increase the risk of early death and frailty, making it an urgent issue to address (13, 14). Consequently, addressing the needs of isolated older adults who struggle to receive family support is a substantial global challenge, and its importance is expected to grow.

In this context, learning from approaches employed to support isolated older people after the Great East Japan Earthquake provides insights for addressing challenges in rapidly aging regions. Prolonged evacuations following the 2011 Great East Japan Earthquake, tsunami, and nuclear accident exacerbated the isolation among older people, adversely affecting their health. There was a subsequent rise in the occurrence of chronic diseases and an elevated risk of receiving care certification (15–17). As the care demands increased, many older people, particularly those without supportive family members, often needed to be placed in managed care facilities, because it is more difficult for them to continue living in their original homes. This concern became especially relevant in areas affected by the 2011 disaster. Therefore, identifying a suitable living environment for isolated older people with or without the need for caregiver assistance remains a persistent issue in the aftermath of long-term evacuation scenarios.

One initiative aimed at addressing the long-term living needs of isolated older people following the Great East Japan Earthquake was the establishment of *Idobata-Nagaya*, a shared living housing model in Soma City. Soma City, which is located 40–50 kilometers from the Fukushima Daiichi Nuclear Power Plant, had a notably high aging rate in 2010, with 25.3% of its population aged over 65 years. The earthquake and subsequent tsunami resulted in 486 deaths in the area, leading more than 2000 people to evacuate to temporary housing (18). The region’s infrastructure and medical system were vulnerable, and the working population later flowed to other regions (19). Older individuals who were self-reliant but had difficulty receiving family support, as well as those who experienced physical decline due to evacuation, wished to stay and continue living in the Soma region. They lived in temporary housing, but their long-term living environment was challenging because it was difficult to obtain support from the community and their families. In 2012, Soma City opened *Idobata-Nagaya*. “*Nagaya*” refers to a traditional architectural style of old Japanese houses (20). “*Idobata*” is a term used to denote a place for socializing. The amalgamation of these two terms led to the creation of “*Idobata Nagaya*” (hereafter *Nagaya*), which was designed as a hub for daily social interaction (21).Within *Nagaya*, residents convene for communal meals, participate in group exercise, monitor each other’s health, and engage in spontaneous interactions (15, 21). The overarching vision of *Nagaya* was to cultivate a community wherein independent older individuals could either delay the need for assisted care or remain even if their health deteriorated. Evaluating the long-term impact of *Nagaya* can contribute to addressing housing issues for older individuals in the context of future disasters.

In this study, we aimed to elucidate characteristics of individuals who were deemed suitable to reside in *Nagaya*, a pioneering collective housing model, following the Great East Japan Earthquake. We also aimed to examine the sustainability of their residency and changes in their care needs over time. We hypothesized that the environment of *Nagaya* reduces the necessity for care for its residents, thus promoting longer durations of stay. By assessing the length of stay and care requirements, this research provides insights into how such housing models can address social isolation, particularly after disasters. Ultimately, we strived to contribute to the broader efforts of mitigating social isolation and enhancing the well-being of the older adult in our aged society.

## 2 Materials and method

### 2.1 Background and participants of *Nagaya*

This was a retrospective cohort study. The participants were those who moved to *Nagaya* in Soma City, Fukushima Prefecture, between May 2012 and December 2022. Between May 2012 and May 2013, five row houses were successively inaugurated, yielding 58 living spaces. At the time of admission, the residents had to be able to live independently and were unable to continue living elsewhere because of the earthquake’s aftermath. To decide who will live in *Nagaya*, city employees conducted individual interviews with each potential inhabitant. The criteria used to determine who should reside in *Nagaya* were their chances of rebuilding their lives, the presence of family support, financial situation, age, any underlying illnesses, and the level of care needs (22).

### 2.2 Services provided in *Nagaya*

*Nagaya* was designed based on the assumption that independent older adults live together. The design of the building is a row house (Supplementary Figures S1, S2). In addition to two bedrooms and an eat-in kitchen room for everyone, a communal bath was installed, and a shared washing machine was provided. The city provides the following services to support daily life: (1) Lunch delivery: an external service delivers lunch boxes daily; (2) a health center nurse checks the residents’ health status every 1–2 months; (3) a transportation service to nearby commercial facilities is provided regularly by bus for shopping purposes; and (4) a contracted caretaker monitors *Nagaya* regularly. Each *Nagaya* elects a delegate to oversee and maintain it autonomously. The representative coordinates lunch with the city’s health department and monitors people’s health.

### 2.3 Data used for analysis

The data used in this study were obtained from Soma City. The anonymized data included information on the residents’ sex, age, underlying diseases, moving-in period, reasons for leaving *Nagaya*, and long-term care insurance system-determined care levels at the start of living and during their stay. In Japan’s long-term care insurance system, care levels are determined based on activities of daily living and physical function. Requiring Long-term care certification starts with requiring support levels 1 and 2 (mild disability) and requiring long-term care levels 1 and 2 (moderate disability) and goes up to requiring long-term care levels 3–5 (severe disability), with a total of 7 stages (23). Older people certified as requiring support levels 1 or 2 can live independently but require some daily living assistance, whereas those certified as requiring long-term care levels 1 or 2 require more assistance. Furthermore, older people with a requiring long-term care level of 3 or higher require constant care and need to move to facilities such as nursing homes.

### 2.4 Statistical analysis

We investigated (1) residents’ characteristics; (2) retention status in *Nagaya* after moving in, including the timing and reasons for leaving; and (3) changes in requiring support/long-term care levels over time. To assess retention, the number of years from moving in to a departure event was calculated by Kaplan–Meier curves. Regarding care level changes, residents without care level certification and those with certified requiring support levels at the time of moving in were targeted. Kaplan–Meier curves were calculated using years until a requiring long-term care level of 1 or higher certification event. Censorship occurred when residents died or left *Nagaya*. Only those who turned 65 years of age during the study period were included in the care-level change analysis since those under 65 years of age have different care certification requirements. This study was approved by the Ethics Review Committee of the Fukushima Medical University (approval number 2022-208). As this was an anonymized, information-based study, research consent was obtained using an opt-out approach.

## 3 Results

### 3.1 Background of the residents

After *Nagaya* began operations in April 2012, 65 people moved into *Nagaya* (Table 1). The average age at the time of moving in was 76.2 years (standard deviation [SD]: 12.0 years), with one person (1.5%) in their teens, six (9.2%) in their 50s, seven (10.8%) in their 60s, 18 (27.7%) aged 70 or older, 29 (44.6%) aged 80 or older, and four (6.2%) aged 90 or older.

**Table 1:** Characteristics of the residents.

Characteristic	Cohort (*n* = 65)
Age at starting residence, mean (SD)	76.2 (12.0)
Age at starting residence, *n* %	
<50 years	1 (1.5)
50–59 years	6 (9.2)
60–69 years	7 (10.8)
70–79 years	18 (27.7)
80–89 years	29 (44.6)
>90 years	4 (6.2)
Female, *n* %	46 (70.8)
Year at starting residence, *n* %	
2012	21 (32.3)
2013	27 (41.5)
2014	7 (10.8)
2015	8 (12.3)
2016	1 (1.5)
2017	0 (0)
2018	0 (0)
2019	1 (1.5)
Certification for requiring long-term care/support needs at starting residence, *n* %	
Without certification	43 (66.2)
Requiring support level 1	6 (9.2)
Requiring support level 2	4 (6.2)
Requiring long-term care level 1	3 (4.6)
Requiring long-term care level 2	4 (6.2)
Requiring long-term care level 3	2 (3.1)
Requiring long-term care level 4	2 (3.1)
Requiring long-term care level 5	1 (1.5)
Residence years, mean (SD)	6.39 (3.83)

SD, standard deviation.

### 3.2 Follow-up results: duration of stay and outcomes

**Figure 1:**
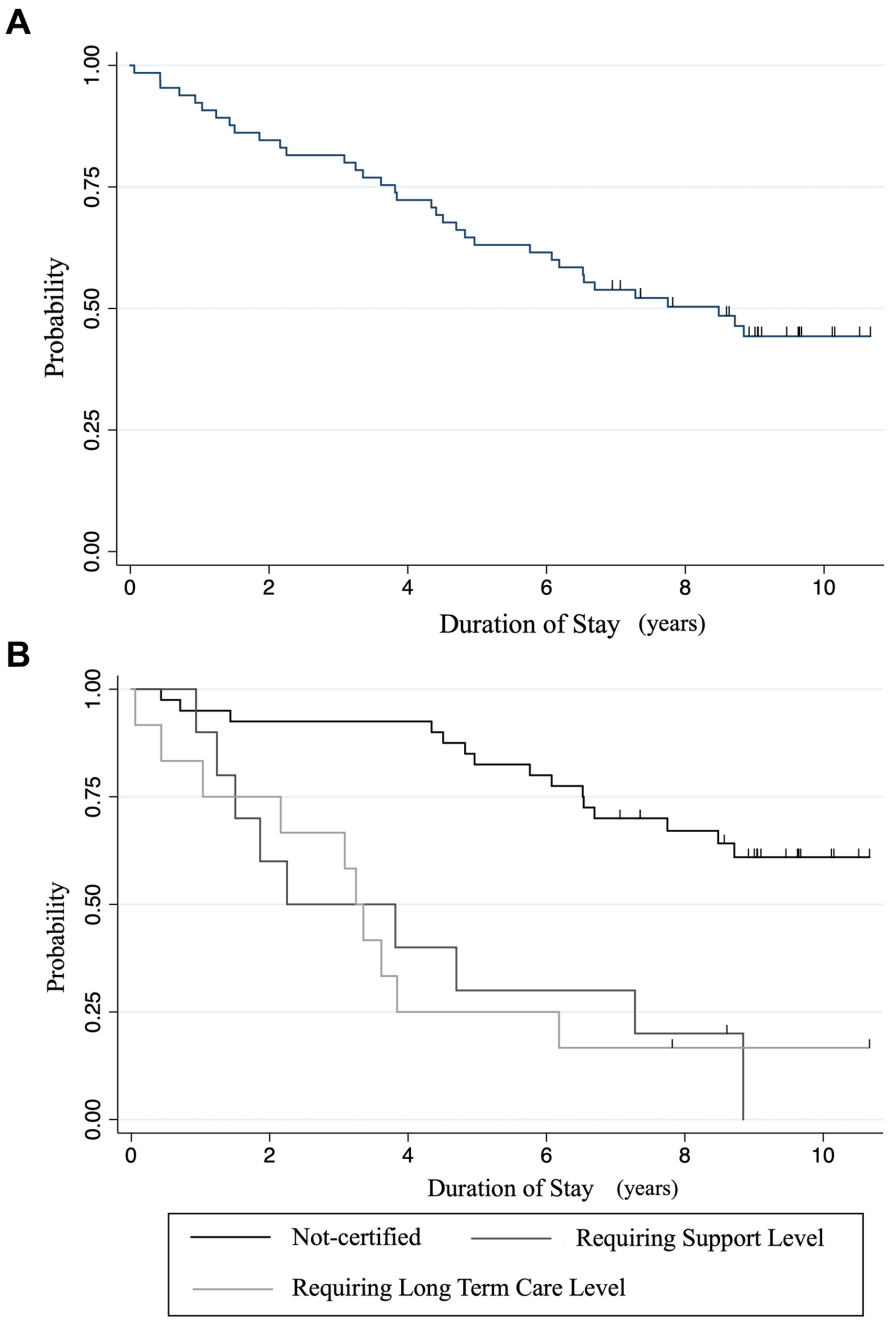
**(A)** Residence retention rates: a Kaplan–Meier survival analysis. **(B)** Residence retention rates by initial care level requirement: a Kaplan–Meier survival analysis. Discontinuation of residence occurred in cases of death, admission to other facilities, relocation to a family member’s home, or departure. Censoring was only performed at the end of the observation period. “Support levels” means “requiring support levels.” “Care levels” means “requiring long-term care levels”.

Of the 65 people, 30 (46.2%) continued to reside in *Nagaya* until December 31, 2022; 21 (32.3%) died during their stay; and the remaining 14 (21.5%) left *Nagaya*. The main reason for leaving was moving to live with relatives (eight people), followed by health-related hospitalization or relocation to a facility (five people). The average overall duration of stay was 6.39 years (SD: 3.83 years), with a median of 7.04 years (interquartile range [IQR]: 3.82–9.08 years), ranging from 21 days to 10.7 years. Regarding the duration of stay, five people left within one year; four people died during the period, and one person left for admission to another facility. Six people left within three years, four people died during this period, and two people moved to live with relatives. Eleven people left within 5 years, with four dying during the study period, four moving to live with relatives, and three leaving for facility admission. The remaining 39 individuals continued to reside for over 5 years. Figure 1A depicts the Kaplan–Meier curve for residence discontinuation. The retention rate declined steadily. Figure 1B shows the Kaplan–Meier curve for residence discontinuation by requiring support/long-term care level at move-in. Details for each age group are provided in the Supplementary material.

### 3.3 Changes in requiring support and long-term care levels

**Figure 2:**
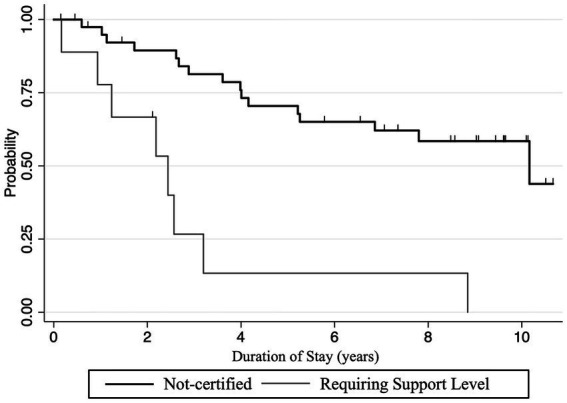
Kaplan–Meier survival analysis of transition to requiring long-term care levels after starting the residence. An event was defined as a newly certified event requiring long-term care after moving in. Censoring was performed at the end of the observation period or at discontinuation of residence.

At admission, 40 of the 62 people who were 65 years and older during the observation period had no requiring support/care certification, 10 had a requiring support level, and 12 had a requiring long-term care level (three with requiring long-term care level 1, four with level 2, two with level 3, two with level 4, and one with level 5) (Table 2). The annual rate of requiring long-term care-level certification was 0.0577 per person-year for the group without requiring support/care certification and 0.3358 per person-year for the group with requiring support-level certification at admission. Figure 2 shows the Kaplan–Meier curve for care-level certification by requiring support/long-term care level at move-in.

**Table 2:** Characteristics regarding requiring long-term care/support levels at the time of starting the residence.

	Total (*n* = 62)	Requiring long-term care/support level at starting residence		
		Not-certified (*n* = 40)	Support level 1 or 2 (*n* = 10)	Long-term care level 1 to 5 (*n* = 12)
Age at starting residence, mean (SD)	77.8 (8.9)	75.9 (8.7)	83.6 (5.1)	79.3 (9.8)
Age at moving out, mean (SD)	84.1 (8.4)	83.4 (8.3)	87.8 (5.0)	83.2 (9.7)
Female, *n* %	45 (72.6)	30 (48.4)	8 (80.0)	7 (58.3)
Residence years, mean (SD)	6.32 (3.28)	7.70 (2.62)	4.10 (2.95)	3.79 (3.0)
Incidence rate for requiring long-term care level 1 or above (per person-years)	–	0.0577	0.3358	–
Current status of the residents^a^, *n* %				
Continued occupancy	28 (45.2)	25 (62.5)	1 (10.0)	2 (16.7)
Deceased	21 (33.4)	9 (22.5)	6 (60.0)	6 (50.0)
Relocation	8 (12.9)	5 (12.5)	2 (20.0)	1 (8.3)
Admitted to another facility	5 (8.1)	1 (2.5)	1 (10.0)	3 (25.0)

SD, standard deviation.

a Status as of December 31, 2022.

Supplementary Tables S1A–C summarize the status of long-term care certifications at 1, 3, and 5 years after admission. Although requiring support/long-term care levels generally increased over time, three people improved their care level during their stay in *Nagaya*; one resident started at requiring long-term care level 3 but improved to requiring long-term care level 2 after 2 years. One individual was certified as requiring long-term care level 2 at move-in, requiring long-term care level 1 a year later, and requiring support level 2 after 2 years. At move-in, the last person had requiring long-term care level 2 but was certified as requiring long-term care level 1 a year later.

## 4 Discussion

We observed a cohort of residents in *Nagaya,* disaster collective housing, 11 years after the community was established. The community welcomed not only independent older people but also those who needed care or were very old and struggling to live alone. Many residents continued living in the community for a long period, and the duration from moving to requiring care was maintained for an extended period. The study suggests that the *Nagaya* environment encourages residents to interact with the community, enabling many residents to maintain their health and continue their independent daily lives. The insights gained in this study can be applied to housing issues for isolated older individuals after other disasters and may also be relevant to housing issues for older isolated individuals in aging societies.

The public housing community initiative contributed to the continued living of older individuals from diverse backgrounds in the Soma region following the disaster. A characteristic challenge in disaster-stricken areas, including the Soma region, is the emergence of diverse housing needs for a wide range of older individuals in terms of age, daily life independence, and frailty. This study revealed variations in age and independence levels among the residents, highlighting the importance of accommodating a diverse older population. Unlike traditional residential facilities with constant professional supervision, such as nursing homes, *Nagaya* fostered an environment for residents to interact and support each other (21). Unlike facilities with strict admission conditions based on care needs (24, 25), *Nagaya* embraced residents with varying levels of frailty and care requirements. While initially designed for independent older individuals, *Nagaya* unintendedly accommodated those requiring mild care, enhancing their quality of life. Although this study did not evaluate social formations among the residents themselves, by creating a supportive environment and promoting social interactions, *Nagaya* may have catered to the needs of older individuals with diverse backgrounds and contributed to their continued living arrangements in the region. The success of *Nagaya* demonstrates the potential of community-based solutions to address the complex care needs of older adults, especially in post-disaster scenarios.

Residents of *Nagaya* have demonstrated the ability to maintain their physical health and reside in the community for extended periods. While direct comparisons with previous studies are challenging, the outcomes appear favorable. For example, in a Japanese cohort study, 44.5% of 746 individuals requiring support level received care level certification within 2 years (26). In another Japanese cohort study, 4.1–4.3% of those requiring support levels transitioned to requiring long-term care level 3 or higher within 3 years, which aligns with the results of this study (27). Notably, given that more than 50% of the residents were aged 80 years or older, the results may be even more promising than those of previous studies. Interestingly, three of the residents in this cohort also showed improvement in their requiring support/long-term care level during their stay, suggesting that the environment in *Nagaya* contributed to reducing the level of care. The presence of a supportive community might have contributed to residents maintaining their independence and preventing the deterioration of care needs (15). Older people participating in social activities have a lower risk of functional disability and death (28, 29), and promoting social activities, including friend interactions, has been suggested to prevent the worsening of care levels (26). Additionally, prioritizing the independence of older adults can have positive impacts on their physical health (7, 30). Communities like *Nagaya* have the potential to uphold physical well-being and delay the transition from frailty to the need for extensive care for independent older individuals.

A notable finding of this study is that many older residents, including those who were very old or certified in care, were able to continue living in *Nagaya* for an extended period. Socially and physically vulnerable individuals may particularly benefit from living in *Nagaya*. After the disaster, those with higher care needs and those living alone are more likely to be negatively affected by changes in their living environment (17). Many residents of *Nagaya* did not have close family members to support them; therefore, their health conditions might have deteriorated more if they had lived alone outside *Nagaya*. On the other side, there were also cases for which *Nagaya* was not well-suited for residents. Five residents left *Nagaya* within a year owing to disease, suggesting that people at high risk for disease exacerbation should consider that *Nagaya*-style facilities are not suitable for them and that they need more specialized care.

Housing facilities such as those in *Nagaya* may become increasingly necessary in the aftermath of large-scale disasters. Problems that may arise after such disasters include the disappearance of living areas for older people living alone and difficulties maintaining a healthy life expectancy. In Hurricane Katrina, those who needed social and medical support had higher mortality rates, and there were reports of increased mental health problems after the disaster (31, 32). Establishing collective housing like *Nagaya* in disaster-stricken areas could help address these problems. Additionally, providing a suitable living environment for older people after a large-scale disaster is essential for facilitating the return of victims to affected areas. In regions hit by the 2011 disaster, there were cases in which returning to residential areas was difficult because of a lack of appropriate care facilities for older people (33). In Katsurao Village, located 20–30 km from the nuclear power plant, difficulty in accessing continuous long-term care insurance services made settling challenging (34). By contrast, in Kawauchi Village, also 20–30 km from the nuclear power plant, the presence of a special nursing home for older adults allowed residents and their families to return to their original homes (35). In one case, a school building was renovated into a care facility to fill its shortage (36). The widespread availability of adequate care facilities in these areas may contribute to the return of the evacuees.

### 4.1 Future implications

Further research is needed to determine the applicability of *Nagaya*-style housing in other disaster scenarios. This study primarily focused on residents’ continuity of residence and care progression. Ongoing interviews and health surveys with residents are crucial for understanding the specific advantages of this housing model. We assume that the results presented herein are due to the formation of social networks by residents. However, it is also important to measure actual participation in social activities. While *Nagaya* showcases potential for broader application in aging communities, further investigation is required. Some residents showed improvement in their level of care during their stay; however, there were scattered cases of rapid progress in the level of care. Future work is needed to determine what factors are associated with maintaining or improving the level of care in a *Nagaya*-like setting. Given the diverse needs arising from different disasters and regions, the universal effectiveness of this housing approach may vary. Examining cases in different cultural contexts is essential to assess the suitability of *Nagaya*-style projects.

### 4.2 Limitation

This study has several limitations. First, it is based on a single case study, which may limit the generalizability of the findings to other contexts or types of disasters. In addition, to highlight the benefits provided by *Nagaya*, comparisons with residents residing in other facilities are desirable. Second, the study primarily focused on the continuity of residence and progression of care needs without considering other factors that might influence the overall well-being and satisfaction of older residents in *Nagaya*-style housing. Third, residents who died during the occupation of *Nagaya* are separated into two groups: those who died in *Nagaya* and those who died in the hospital, the details of which remain unclear. Lastly, this study did not measure individual-specific factors, such as physical activity levels, functional abilities, or detailed medical histories of older residents. It should be noted that this is a data-based analysis and does not directly measure indicators of health.

## 5 Conclusion

Soma *Nagaya* initiatives showed the potential to provide a suitable living environment for older individuals with diverse needs in the aftermath of disasters. These findings suggest that *Nagaya*-style housing could be a useful tool for solving the housing concerns of isolated older individuals in disaster-prone areas.

## Data availability statement

The datasets presented in this study can be found in online repositories. The names of the repository/repositories and accession number(s) can be found in the article/Supplementary material.

## Ethics statement

The studies involving humans were approved by Fukushima Medical University Ethics Review Committee Chair, Clinical Research Review Committee for Guidelines Chair, General Ethics Committee. The studies were conducted in accordance with the local legislation and institutional requirements. Written informed consent for participation was not required from the participants or the participants’ legal guardians/next of kin in accordance with the national legislation and institutional requirements.

## Author contributions

TA: Conceptualization, Data curation, Formal analysis, Writing – original draft, Writing – review & editing. HS: Conceptualization, Data curation, Formal analysis, Writing – original draft, Writing – review & editing. NM: Writing – review & editing. NI: Writing – review & editing. MoT: Writing – review & editing. YK: Writing – review & editing. AO: Writing – review & editing. YN: Writing – review & editing. CY: Writing – review & editing. TZ: Writing – review & editing. MS: Writing – review & editing. MaT: Funding acquisition, Project administration, Resources, Supervision, Writing – review & editing.
