# Supplementary material for: Idobata-Nagaya: a community housing solution for socially isolated older adults following the great East Japan earthquake
Source: Front Public Health. 2023 Nov 22;11:1289552. doi: 10.3389/fpubh.2023.1289552 (PMC10703167; doi:10.3389/fpubh.2023.1289552)
Supplement: Supplementary file 1 [file Data_Sheet_1.pdf]

Supplementary Material

1 Supplementary data

Follow-up results: duration of stay and outcomes by age groups

Regarding the age groups, one person under 50 years old moved away, and of the six people in their 50s, all (100%) continued to reside until December 31, 2022. Of the seven people in their 60s, four (57%) continued to reside, two (28%) died during the study period, and the remaining one (14%) was admitted to another facility. Six people continued to reside there for over one year and five people for over five years. Of the 18 people in their 70s, 12 (67%) continued to reside, five (28%) died during the period, and the remaining one (14%) moved to live with relatives. All 18 people continued to reside for over one year and 15 people for over five years. Of the 29 people in their 80s, eight (44%) continued to reside, while 12 (67%) died during their stay in *Nagaya*. Of the remaining nine (50%), six moved to live with relatives, and three were admitted to another facility. Twenty-five people continued to reside there for over one year and 15 people for over five years. Of the four people in the 90s, two died during their stay, one moved to live with relatives, and one was admitted to another facility.

2 Supplementary Figures and Tables

2.1 Supplementary Figures

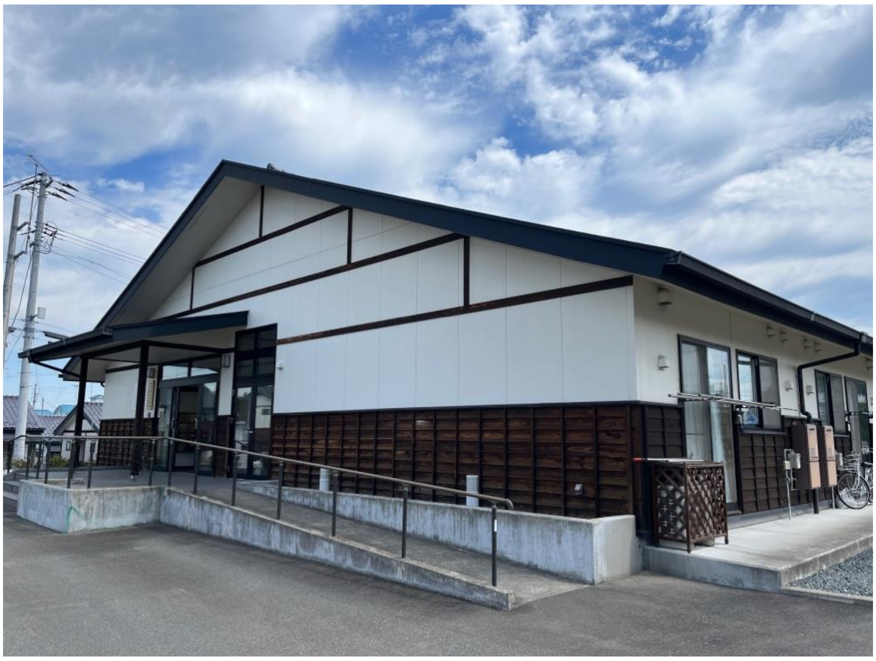

Supplementary Figure 1. During the Edo period, this architectural style, called *Nagaya*, was commonly used by common people in the downtown area.

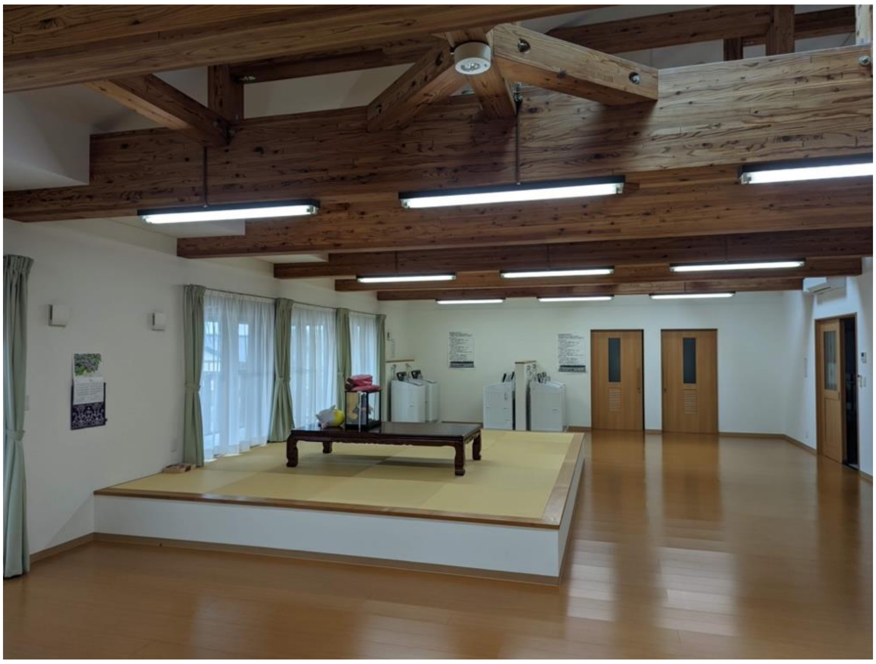

Supplementary Figure 2. Shared community lounge in *Nagaya.* It is designed as a space where people can interact while using the common laundry machines.

2.2 Supplementary Tables

Supplementary Table 1a Changes in the number of residents certified for requiring long-term care/support needs 1 year after starting the residence

		Requiring Long-term care/support level at starting residence								
		**Not certified (n=38)**	**Support level 1 (n=6)**	**Support level 2 (n=3)**	**Long-term Care level 1 (n=3)**	**Long-term Care level 2 (n=4)**	**Long-term Care level 3 (n=1)**	**Long-term Care level 4 (n=2)**	**Long-term Care level 5 (n=1)**	**Total (n=57)**
**Requiring Long-term care/support Levels at one year after staring residence**	Not certified	34 (89.5)	-	-	-	-	-	-	-	34 (59.6)
	Support level 1	1 (2.6)	4 (66.7)	-	-	-	-	-	-	5 (8.8)
	Support level 2	1 (2.6)	-	3 (100)	-	-	-	-	-	4 (7.0)
	Long-term Care level 1	-	1 (16.7)	-	1 (33.3)	2 (50.0)	-	-	-	4 (7.0)
	Long-term Care level 2	1 (2.6)	1 (16.7)	-	2 (66.7)	1 (25.0)	-	1 (50.0)	-	6 (10.5)
	Long-term Care level 3	-	-	-	-	1 (25.0)	1 (100)	-	-	2 (3.5)
	Long-term Care level 4	1 (2.6)	-	-	-	-	-	-	-	1 (1.8)
	Long-term Care level 5	-	-	-	-	-	-	1 (50.0)	1 (100)	2 (3.5)

Focused on those living in *Nagaya* for more than a year. Numbers represent the count of residents and the proportion of them.

Supplementary Table 1b Changes in the number of residents certified for requiring long-term care/support needs 3 years after starting the residence

		Requiring Long-term care/Support level at starting residence								
		**Not certified (n=37)**	**Support level 1 (n=3)**	**Support level 2 (n=2)**	**Long-term Care level 1 (n=2)**	**Long-term Care level 2 (n=4)**	**Long-term Care level 3 (n=1)**	**Long-term Care level 4 (n=1)**	**Long-term Care level 5 (n=0)**	**Total (n=50)**
**Levels at three years after staring residence**	Not certified	26 (70.3)	-	-	-	-	-	-	-	26 (13)
	Support level 1	2 (5.4)	1 (33.3)	-	-	-	-	-	-	3 (1.5)
	Support level 2	4 (10.8)	-	1 (50.0)	-	1 (25.0)	-	-	-	6 (3.0)
	Long-term Care level 1	2 (5.4)	2 (66.7)	1 (50.0)	-	1 (25.0)	-	-	-	6 (3.0)
	Long-term Care level 2	2 (5.4)	-	-	1 (50.0)	-	1 (100)	-	-	4 (2.0)
	Long-term Care level 3	-	-	-	1 (50.0)	-	-	-	-	1 (0.5)
	Long-term Care level 4	1 (2.7)	-	-	-	1 (25.0)	-	1 (100)	-	3 (1.5)
	Long-term Care level 5	-	-	-	-	1 (25.0)	-	-	-	1 (0.5)

Focused on those who have been living in *Nagaya* for more than three years. Numbers represent the count of residents and the proportion of them.

Supplementary Table 1c Changes in the number of residents certified for requiring long-term care/support needs 5 years after

starting the residence

		Requiring Long-term care/support level at starting residence								
		**Not certified (n=34)**	**Support level 1 (n=2)**	**Support level 2 (n=1)**	**Long-term Care level 1 (n=1)**	**Long-term Care level 2 (n=2)**	**Long-term Care level 3 (n=0)**	**Long-term Care level 4 (n=0)**	**Long-term Care level 5 (n=0)**	**Total (n=40)**
**Levels at five years after staring residence**	Not certified	22 (64.7)	-	-	-	-	-	-	-	22 (55)
	Support level 1	2 (5.9)	1 (50.0)	-	-	-	-	-	-	3 (7.5)
	Support level 2	4 (11.8)	-	-	-	1 (50.0)	-	-	-	5 (12.5)
	Long-term Care level 1	1 (2.9)	1 (50.0)	1 (100)	-	-	-	-	-	3 (7.5)
	Long-term Care level 2	2 (5.9)	-	-	-	1 (50.0)	-	-	-	3 (7.5)
	Long-term Care level 3	2 (5.9)	-	-	-	-	-	-	-	2 (5.0)
	Long-term Care level 4	2 (5.9)	-	-	-	-	-	-	-	2 (5.0)
	Long-term Care level 5	-	-	-	1 (100)	-	-	-	-	1 (2.5)

Focused on those living in *Nagaya* for more than five years. Numbers represent the count of residents and the proportion of them.
